# Genetic Mutations and Variants in the Susceptibility of Familial Non-Medullary Thyroid Cancer

**DOI:** 10.3390/genes11111364

**Published:** 2020-11-18

**Authors:** Fabíola Yukiko Miasaki, Cesar Seigi Fuziwara, Gisah Amaral de Carvalho, Edna Teruko Kimura

**Affiliations:** 1 Department of Endocrinology and Metabolism (SEMPR), Hospital de Clínicas, Federal University of Paraná, Curitiba 80030-110, Brazil; fymiasaki@gmail.com (F.Y.M.); carvalho.gisah@gmail.com (G.A.d.C.); 2 Department of Cell and Developmental Biology, Institute of Biomedical Sciences, University of São Paulo, São Paulo 05508-000, Brazil; cesar.fuziwara@usp.br

**Keywords:** thyroid cancer, thyroid neoplasms, genetic predisposition to disease, genetic variants

## Abstract

Thyroid cancer is the most frequent endocrine malignancy with the majority of cases derived from thyroid follicular cells and caused by sporadic mutations. However, when at least two or more first degree relatives present thyroid cancer, it is classified as familial non-medullary thyroid cancer (FNMTC) that may comprise 3–9% of all thyroid cancer. In this context, 5% of FNMTC are related to hereditary syndromes such as Cowden and Werner Syndromes, displaying specific genetic predisposition factors. On the other hand, the other 95% of cases are classified as non-syndromic FNMTC. Over the last 20 years, several candidate genes emerged in different studies of families worldwide. Nevertheless, the identification of a prevalent polymorphism or germinative mutation has not progressed in FNMTC. In this work, an overview of genetic alteration related to syndromic and non-syndromic FNMTC is presented.

## 1. Introduction

**Figure 1:**
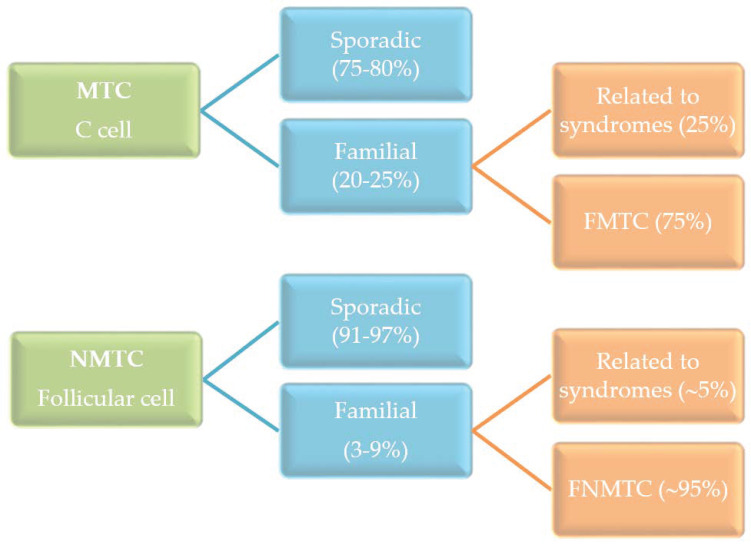
Incidence of sporadic and familial medullary thyroid cancer (MTC) and non-medullary thyroid cancer (NMTC).

The most common type of thyroid cancer derives from thyroid follicular cells and is named as non-medullary thyroid cancer (NMTC) in order to be distinguished from the less frequent medullary thyroid cancer (MTC) that originates from the thyroid C-cells. The MTC occurs as sporadic and hereditary cancer, in contrast to the NMTC, which is mainly sporadic (Figure 1). The hereditary MTC can be a component of a syndrome or have a familial background. In this context, the NMTC can also be associated with syndromic conditions, such as in Cowden syndrome, Carney complex, Werner syndrome, and familial adenomatous polyposis but to a lesser extent than in MTC. Moreover, a high prevalence of NMTC in ataxia-telangiectasia, DICER1, and Pendred syndromes has been described [1,2].

Besides these well-known genetic syndromes, the characterization of the non-syndromic form of familial non-medullary thyroid cancer (FNMTC) remains to be consolidated. In 1953, Firminger and Skelton reported the first case of papillary thyroid cancer (PTC) in twins [3]. However, the concept of FNMTC and the genetic predisposition to PTC has emerged only in recent decades. Currently, it is accepted that FNMTC occurs when two or more first-degree relatives are diagnosed with NMTC cancer [4].

The initial FNMTC studies were performed by linkage analysis and described some specific loci, although they did not identify a precise gene associated with FMNTC [5,6,7,8,9,10]. Furthermore, despite the efforts of many groups in investigating FNMTC using Sanger sequencing, no conclusive information was found, suggesting genetic heterogeneity, multigenic inheritance, and multifactorial inheritance [11]. However, a new genomic perspective emerged with the application of Next Generation Sequencing (NGS) technology that covered the entire genome. In this extent, some new insights into genetics of FNMTC have emerged by the recent genome-wide association studies (GWAS) in populations of PTC. The finding of several single nucleotide polymorphisms (SNPs), such as in *DIRC3*, *NIRG1*, *FOXE1*, *NKX2-1*, and *PCNXL2*, were observed in the European, Korean, and American populations [12]. Increasing evidence suggests that genetic predisposition factors play an essential role in carcinogenesis besides environmental factors [13]. In this review, we cover the genetic findings associated with FNMTC and in syndromes related to NMTC.

## 2. Syndromic Causes of Non-Medullary Thyroid Cancer

Many syndromes associated with thyroid tumor predisposition have Mendelian patterns of inheritance, and they are related to mutations that may influence the mechanism of DNA repair, the microRNA processing, and maturation, the genome integrity maintenance, the cell signaling, or mitochondrial regulated cellular processes (Table 1) [13]. These syndromes are characterized by several other main malignancies and sometimes lack to present thyroid cancer. Some authors have suggested surveillance in syndromic FNMTC (see below). However, the guidelines, such as the 2015 American Thyroid Association’s guideline, are precautious due to insufficient evidence in order to recommend the thyroid cancer screening [14].

### 2.1. Cowden Syndrome

Cowden syndrome (OMIM #158350) is characterized by hamartomas in different parts of the body (gastrointestinal hamartomas, ganglioneuromas, trichilemmomas) associated with melanomas, breast, endometrial and thyroid cancer, macrocephaly, and, eventually, autism spectrum disorder and/or mental retardation [18,28]. One of the major diagnosis criteria is the presence of follicular thyroid carcinoma (FTC). However, if it is not detected, a biannual thyroid ultrasound is advocated in patients older than seven years [29].

Cowden syndrome is classically associated with mutations in the phosphatase and tensin homolog (*PTEN*) gene on chromosome 10q22–23, although variants in several other genes have been described in patients without *PTEN* mutations (*SDHB-D, SEC23B, KLLN, PARP4, AKT1, PIK3CA, USF3, TTN, MUTYH, RET, TSC2, BRCA1, BRCA2, ERCC2, HRAS*, and *RASAL1*) [19,30,31,32].

PTEN dephosphorylates PI (3,4,5) P3 (PIP3) and PI (3, 4) P2 to PI (4,5) P2 and PI (4) P, respectively. Thus, PIP3 and PI (3,4) P2 do not activate AKT serine/threonine kinase (AKT), and the phosphatidilinositol 3-kinase (PI3K)/AKT signaling pathway remains inhibited. PTEN mutation results in loss of function, leading to a high concentration of PI (3,4) P2 that activates AKT and enhances cell proliferation, cell migration, and reduces cell death [33,34]. In addition, mechanisms that regulate PTEN expression and compartmentalization are involved in tumorigenesis [35].

The first correlations between the PIK3-AKT pathway activation and thyroid cancer were observed in Cowden syndrome studies. Since Cowden syndrome mainly presents with FTC and PTEN activates the PIK3-AKT pathway, some authors have postulated that PIK3-AKT activation is required for FTC oncogenesis, and these preliminary findings were further corroborated [36,37].

Another intriguing fact was the association of RAS protein activator like 1 (*RASAL1*) with Cowden Syndrome. RASAL1 is a negative modulator of the RAS signaling pathway and suppresses both mitogen-activated protein kinase (MAPK) and PI3K pathways. However, *RASAL1* is frequently found methylated or mutated in sporadic follicular and anaplastic thyroid cancer [38].

In a large series of 155 patients with Cowden syndrome and thyroid cancer, 39 presented with *PTEN* germline mutations, while *RASAL1* germline alteration (*RASAL1*, c.982C>T, R328W) was observed in two patients without *PTEN* mutations [32]. In the same study, the authors also analyzed the germline database of The Cancer Genome Atlas (TCGA) and discovered that 0.6% of PTC patients harbored the deleterious germline *RASAL1* mutation [32].

### 2.2. Carney Complex

The Carney complex (OMIM #160980) is an autosomal dominant disorder in 70% of the cases, characterized by loss-of-function mutations in the *PRKAR1A* gene (17q22–24).

Under a normal condition of several endocrine related ligands, such as TSH, FSH, ACTH, GHRH, and MSH, when binding to the G-protein coupled receptor activates protein kinase A (PKA). *PRKAR1A* encodes the R1α subunit of PKA. Thus, when mutated, it increases cAMP-dependent PKA activity and drives tumorigenesis [17,39,40]. Therefore, thyrocytes, Sertoli cells, adrenocortical cells, somatotrophs, and melanocytes are directly affected by the *PRKAR1a* mutation. As a result, variable endocrine tumors are observed in the Carney complex disease, including primary pigmented nodular adrenocortical disease, pituitary adenomas, testicular tumors, ovarian lesions, and myxomas and lentiginosis syndromes [17]. Since thyroid cancer could also be part of this syndrome, annual long-term surveillance is recommended [17].

Evidence shows that PRKAR1A acts as a tumor suppressor gene in sporadic thyroid cancers [41]. However, the traditional thyroid cancer pathways (MAPK and PIK3-AKT pathways) are not involved in the Carney complex [42]. Instead, a recent in vitro study suggests that PKA activates AMP-activated kinase (AMPK) through serine/threonine kinase 11 (LKB1, also named SKT11) in Carney-related FTC without inhibiting mTOR activation [43].

### 2.3. Werner Syndrome

Werner syndrome is one of the progeroid syndromes (OMIM #27770) characterized by early aging, scleroderma-like skin changes, bilateral cataracts, and subcutaneous calcifications, premature arteriosclerosis, and diabetes mellitus. Different types of cancers are associated with this syndrome, such as meningiomas, myeloid disorders, soft tissue sarcomas, and thyroid carcinoma [1,44]. Their regular surveillance is recommended [45]. The Werner Syndrome’s patients carry autosomal recessive WRN RecQ like helicase (*WRN*) gene mutations on 8p11.1–21.1. *WRN* gene encodes RecQ helicase that regulate DNA replication, recombination, repair, transcription, and telomerase maintenance. Dysregulation of this pathway triggers DNA instability, telomeric fusions of homologous chromosomes, and, ultimately, oncogenesis [13]. However, the precise mechanisms that contribute to genome instability in Werner syndrome remains unclear [46]. In a Japanese series, mutations in the N-terminal portion of *WRN* was correlated with PTC, while mutations in C-terminal with FTC [47]. The N-terminal portion of *WRN* contains exonuclease activity, whereas the central part contains the DNA-dependent ATPase, 3′–5′ helicase, and annealing activity [46]. Overall, these studies suggest specific effects in WRN activity depending on the site of mutation. Moreover, an in vitro study showed that mutations in *WRN*’s nuclease domain, helicase domain, or DNA binding domain aborted its canonical stimulatory effect on nonhomologous end-joining (c-NHEJ) pathway during DNA double-strand break (DSB) repair [46].

### 2.4. Familial Adenomatous Polyposis

The phenotype of familial adenomatous polyposis (FAP) (OMIM #175100) is characterized by numerous intestinal polyps, colon cancer, and other cancers that include thyroid cancer [2,22,48]. FAP is an autosomal dominant disorder caused by mutations in APC regulator of WNT signaling pathway (*APC*) gene on chromosome 5q21. The *APC* gene is a suppressor of the Wnt signaling pathway and regulates *β*-catenin activation by multiple mechanisms. In normal conditions, the Axin complex—formed by APC, glycogen synthase kinase 3 (GSK3), and casein kinase 1 (CK1)—phosphorylates the amino-terminal of the free *β*-catenin, permitting its recognition and further ubiquitination [49,50]. By this process of continuous degradation, *β*-catenin remains in the cytoplasm without reaching the promoter region of target genes in the nucleus. Thus, when the APC protein is mutated or truncated, *β*-catenin is released from its degradation and migrates to the nucleus, activating gene transcription of oncogenic pathways. Truncated APC protein also interferes with chromosome stability and cell migration [50].

In addition to the germline mutation, biallelic inactivation of the wild-type APC allele is frequently necessary for tumorigenesis, and the second-hit is commonly acquired by somatic mutation [51]. In the FAP-associated thyroid cancer, the concomitant presence of germline and distinct somatic mutation were observed in several Japanese families [48,52,53]. Most of FAP-associated thyroid cancers present the histological subtype called cribriform-morular variant of PTC (CMVPTC) [2,51]. An annual thyroid ultrasound is recommended to late teen years’ patients [54,55].

### 2.5. Ataxia-Telangiectasia Syndrome

Ataxia-telangiectasia (A-T) syndrome (OMIM #208900) is an autosomal recessive disorder linked to the mutation of the ATM serine/threonine kinase (*ATM*) gene and characterized by degenerative cerebellar atrophy, telangiectasias, immune defects, and malignancy [56,57]. It is also well-known that relatives of patients with ataxia-telangiectasia have an increased cancer incidence [16].

ATM protein belongs to the PI-3 kinase-like protein kinases family. Besides TP53, BRCA1, and BRCA2, ATM is considered a genome’s guardian and participates directly in the DNA damage response (DDR). For its activation, MRE11-RAD50-NBS1 (MRN) complex—A sensor of DSB (double strand-break)—induces several autophosphorylations and acetylations. Activated ATM then phosphorylates different proteins involved in the DSB (double-strand break) response [58]. For instance, ATM phosphorylates CHK2 and p53, which are both involved in senescence and apoptosis [58].

An increased incidence of thyroid cancer was observed in obligate *ATM* mutation carriers (RR adjusted = 2.6) [16]. Later, selective mutations in the *ATM* gene are related to thyroid cancer. *ATM* c.2119T>C p.S707P (rs4986761) heterozygotes were associated with an adjusted HR (hazard ratio for cancer) of 10 for thyroid/endocrine tumors, while no association was observed in *ATM* c.146C>G p.S49C (rs1800054) heterozygote carriers [56]. Nonetheless, recent population studies revealed that some ATM polymorphisms have a protective role, while other studies reported a damaging effect [59,60,61,62]. There are even controversial observations for the same polymorphism [26,27,59,60,63]. Despite these controversies, consistent *ATM* variants (*ATM* p.P1054R-rs1800057- and rs149711770) were recently described in families with FNMTC and other cancers (as kidney, lung, stomach, and prostate) [11]. Nonetheless, in A-T Syndrome´s patient, the only screening recommended is for breast cancer [15].

### 2.6. DICER 1 Syndrome and miRNA Processing

A non-toxic multinodular goiter (MNG) is frequently diagnosed in the adult population and studies correlate the presence of MNG and the development of differentiated thyroid cancer [14,64,65]. On the other hand, familial cases of MNG are a common characteristic associated with the DICER1 syndrome (OMIM #601200), which predisposes patients to thyroid cancer [66], and other types of tumors such as Sertoli-Leydig cell tumors of the ovary (SLCT) [20] and pleuropulmonary blastomas [21]. 

DICER is an endonuclease essential for the maturation of microRNAs (miRNAs), small non-coding RNAs with ~22 nt, that block mRNA translation post-transcriptionally by binding to the 3′-UTR (untranslated region) of target mRNAs, and tightly controlling cell signaling and cell biology [67]. A mutation in dicer1, ribonuclease III (*DICER1*) gene, especially those present in the ribonuclease domain, leads to DICER loss of function and downregulation of microRNA levels [20,68]. The correct control of miRNA expression is essential for the development of a functional thyroid gland [69]. Studies with transgenic mice with dysfunctional DICER lead to disturbance of thyroid architecture, cell proliferation and disarrangement of follicular structures, and loss of differentiation [70,71], indicating the influence of DICER loss in thyroid tumorigenesis.

A familial approach to investigate the risk of thyroid malignancy in DICER1 syndrome patients revealed a 16-fold higher risk of development of thyroid cancer when *DICER1* is mutated compared to non-mutated patients [66]. Thus, there is a suggestion to monitor the thyroid status by a thyroid ultrasound every two-three years in patients after the age of eight [29,72]. Enforced evidence of *DICER1* mutation with familial thyroid cancer was also shown in a study with six individuals of the same family harboring *DICER1* mutation (c.5441C>T, p.S1814L) and multiple cases of differentiated thyroid cancer and MNG [73]. 

The Cancer Genome Atlas (TCGA) database shows *DICER1* mutation in 0.8% of patients with PTC/PDTC (p.E1813G, p.D1810H, p.E1813K, p.R1906S, p.M1402T) [74,75,76,77,78]. A recent study revealed high prevalence of *DICER1* mutations in pediatric-adolescent poorly differentiated thyroid cancer (83%) at a hotspot in the metal-ion binding sites of the RNase IIIb domain of DICER1 (c.5113G>A, p.E1705K, c.5125G>A, p.D1709N (rs1595331264), c.5137G>A, p.1713Y, c.5437G>A, p.E1813K, c.5437G>C, p.E1813Q) [79]. Another study linked hotspot *DICER1* mutations to pediatric PTC (c.5125G>A p.D1709N, c.5428G>T p.D1801Y, c.5438A>G p.E1813G, c.5439G>C p.E1813D) with increased incidence in the patients that do not harbor MAPK classic alterations [80], suggesting a role for *DICER1* mutation detection in thyroid tumors. A recent study detected *DICER1* (c.5429A>T, p.D1810V, c.5437G>A, p.E1813K) and drosha ribonuclease III (*DROSHA*) mutation (c.2943C>T, p.S981S, c.3597C>T, p.Y1199Y (rs61748189)) in benign follicular adenoma, even though *DICER1* mutations were not detected in a follicular variant of PTC that harbored *HRAS* mutations [68]. On the other hand, a recent study associated MAPK alterations with germline mutations in *DICER1* [81]. Altogether, these studies suggest that *DICER1* haploinsufficiency is associated with thyroid tumorigenesis.

DROSHA is another endonuclease of miRNA processing machinery and acts together with DGCR8 to form the Microprocessor complex to excise the precursor miRNA out of the primary transcript in the nucleus [67]. Then, DICER acts in the next step in the cytoplasm and cleaves the precursor miRNA to form mature functional miRNAs. In a similar extent to *DICER1* mutations, *DGCR8* mutations were also detected in familial cases of MNG and are associated with schwannoma [82]. Altogether, these studies indicate the essential role of proper miRNA processing and expression for thyroid gland physiology.

### 2.7. Li-Fraumeni Syndrome

The Li-Fraumeni syndrome is caused by a heterozygous mutation in *TP53* and is typically characterized by soft tissue and bone sarcomas, breast cancers, central nervous system tumors, leukemia, and adrenal tumors. p53 interacts with a complex network and drives DNA repair, cell-cycle arrest, senescence, or apoptosis when it is phosphorylated by DNA damage response (DDR) kinases [13,83]. The PTC occurs in 10% of Li-Fraumeni syndrome patients, mainly when associated with *TP53* mutation p.R337H [24]. Therefore, imaging screening for thyroid malignancy in Li-Fraumeni families has been advocated [24].

## 3. Non-Syndromic FNMTC

Even if FNMTC comprises only 3–9% of all thyroid cancer, the first-degree relatives of NMTC have an 8-12-fold increased risk of developing the disease [84,85]. Non-syndromic FNMTC comprises 95% of all FNMTC and is defined by two or more first-degree relatives present with NMTC without associated syndromes. Moreover, the transmission pattern is not yet well defined, which seems to be autosomal dominant in most cases. Like sporadic NMTC, more than 85% are PTC, approximately 10% are FTC, and around 5% are anaplastic thyroid cancer. Furthermore, FNMTC is more aggressive, presents with nodal disease, and recurs more often. In addition, thyroid cancer tends to occur earlier in subsequent generations in FNMTC, called the anticipation phenomenon [2,86,87].

### 3.1. Linkage Analysis

From 1997 to 2006, the linkage analysis was the main method to study the familial condition. Using this approach, a positive logarithm of odds (LOD) would mean a high likelihood that locus cosegregates with the FNMTC trait, which is a linkage. In this way, several loci were associated with non-syndromic FNMTC (Table 2).

#### 3.1.1. TCO Locus (19p13.2)

The ‘thyroid carcinoma, nonmedullary, with cell oxyphilia’ (TCO) locus was identified in a French family with oxyphilic thyroid cancer in the short arm of chromosome 19 (19p13.2). This region includes several genes, such as *ICAM1* gene, which is overexpressed in thyroid cancer cells, and the JunB proto-oncogene, AP-1 transcription factor subunit (*JUNB*) [6]. However, some other genes in the locus, such as several zinc-finger-protein genes, were not yet identified. Moreover, the TCO locus does not seem to be involved in the majority of oxyphilic sporadic NMTC. An additional Tyrolean family with high LOD in the same locus was also described [93]. 

#### 3.1.2. PRN1 Locus (1q21)

Papillary thyroid cancer is associated with papillary renal cancer. Linkage analysis identified this locus with the highest LOD of 3.58 in a family with three generations affected by PTC and papillary renal carcinoma. MET proto-oncogene, receptor tyrosine kinase (*MET*) mutations, frequently associated with familial papillary renal cancer, and mutations associated with other thyroid cancer syndromes were excluded [8]. However, this finding was limited to this family.

#### 3.1.3. NMTC1 Locus (2q21)

This locus was described in a large Tasmanian family study [9], and when the authors further analyzed 17 families with FNMTC, they found an LOD heterogeneity of 4.17. At that time, it was hypothesized that multiple environmental and genetic causes could be involved in the pathogenesis of FNMTC [93].

#### 3.1.4. q32 Locus (an Enhancer of Unknown Function)

A rare mutation in 4q32 was found in the linkage analysis and targeted deep sequencing in a large family with four individuals with benign thyroid disease, nine PTC patients, and one anaplastic thyroid cancer (ATC) patient. This nucleotide exchange in chr4:165491559 (GRCh37/hg19), named 4q32A>C, is in a highly conserved region. The chromatin immunoprecipitation (ChIP) assays showed that both POU2F1 and YY1 transcription factors related to specific thyroid genes and thyroid development bind to this region. As consequence of the allele’s change, a decrease of both POUF2 and YY1 bindings were observed. Transcription factors’ disruption has already been associated with cancer [91].

#### 3.1.5. 6q22 Locus

The finding of 6q22 locus with LOD + 3.30 was observed in 38 families of FNMTC by linkage analysis and a genome-wide SNP array [89]. However, no further studies have confirmed this locus in additional families.

#### 3.1.6. 8p23.1–p22 Locus

A locus associated with FNMTC in a huge Portuguese family was identified by linkage analysis, with a maximum parametric haplotype-based LOD score of 4.41. Among 17 candidate genes in the locus (*PPP1R3B, MIRN597, MIRN124A1, MSRA, C8orf74, SOX7, PINX1, MIRN598, C8orf15, C8orf16, MTMR9, C8orf13, NEIL2, CTSB, DUB3, DLC1, TUSC3*), no deleterious alteration was detected in the genes’ coding region. [10].

#### 3.1.7. 8q24 Locus, a lncRNA inside the Thyroglobulin (TG) Gene

Linkage analysis was also performed in a group of 26 families of PTC [90], which revealed a LOD of + 1.3 in a locus that harbors *TG* and *SLA* (Src like adaptor) genes. However, no polymorphism or mutation was found in the coding genes, suggesting that this alteration could be associated with a lncRNA related to the *TG* gene.

#### 3.1.8. SRGAP1 (12q14 Locus)

The study of 38 families with FNMTC by genome-wide linkage analysis indicated a high peak in 12q14 in 55% (21 of 38), but with a modest OR = 1.21 (*p* = 0.0008). Nonetheless, it was observed six different germline mutations/variants in the *SRGAP1* gene (c.447A>C, p.Q149H, c.823G>A, p.A275T, c.1534G>A, p.V512I, rs74691643, c.1849C>T, p.R617C, rs114817817, c.2274T>C, p.S758S, rs789722, c.2624A>G, p.H875R, rs61754221). In vitro functional testing in thyroid cancer cells showed decreased GTPase activating protein (GAP) activity in two of these *SGARP1* polymorphisms (Q149H and R617C). The SRGAP1 could mediate tumorigenesis by interacting with CDC42 [88], which is a common signal transduction convergence point of many signaling pathways and can play a role in thyroid cancer cell migration via RAGE/Dia-1 signaling [94].

#### 3.1.9. NKX2-1 (14q13.3 Locus)

The mutation in *NKX2-1* gene (c.1016 C>T, p. A339V) was described in two families associated with PTC and MNG [87]. Even though most patients had only MNG, the authors hypothesized that MNG could be the first step to malignancy [92,95,96,97]. 

#### 3.1.10. MNG1 Locus (14q32)-DICER1

The MNG1 (OMIM # 138800) locus was revealed by linkage analysis in families with multinodular goiter and NMTC [7]. Furthermore, it was observed that MNG1 corresponded to *DICER1* gene, related to microRNA biogenesis (described in “Syndromic causes of non-medullary thyroid cancer” section).

### 3.2. Genome-Wide Linkage Analysis in the Population of PTC Patients

The sequencing of the genome by NGS) uncovered the genetic variation and the potential association with several pathologies, including cancer. In particular, the GWAS (genome-wide association study) revealed numerous SNPs in the genes related to thyroid physiology and tumorigenesis (Table 3) [12].

#### 3.2.1. *FOXE1/PTCSC2*

Located in 9q22.3 and close to the forkhead box E1 (*FOXE1*) gene, rs965513 conferred an increased risk for thyroid cancer and was named ‘papillary thyroid carcinoma susceptibility candidate 2’ (*PTCSC2*) gene. The carriers of rs965513 (homozygous of A allele present a 3.1-fold increased risk for thyroid cancer in large European series [98]. The same polymorphism rs965513 was observed in Japanese and Belarusian populations, but with an OR of 1.6-1.9 [104]. Similarly, a variant in the promoter region of the *FOXE1* gene (rs1867277) was identified as a risk factor for PTC (OR = 1.49) in a Spanish series and further confirmed in an Italian one [105]. Subsequently, new studies showed a tumor suppressor effect of FOXE1 and demonstrated that rs1867277 is involved in differential recruitment of USF1/ USF2 transcription factors, which interferes with FOXE1 expression [12,106]. Moreover, myosin heavy chain-9 (MYH9) can bind and suppress the shared promoter of *PTCSC2* and *FOXE1* genes bilaterally (that includes rs1867277 region), an effect that is abolished by PTCSC2 that sequesters MYH9 [107]. Therefore, MYH9, which is a lncRNA binding protein, can also play a role in PTC susceptibility.

A rare *FOXE1* variant (c.743C>G; p.A248G) was identified in one of 60 Portuguese FNMTC cases and one sporadic case. Besides, polymorphisms in *FOXE1* locus (rs965513 and rs1867277) were associated with increased familial and sporadic NMTC risk [104,108].

#### 3.2.2. *NKX2-1*

A consistent finding in the 14q13.3 locus was rs944289. Located close to the NKX2-1 gene, this variant of uncertain significance (VUS) is in PTCSC3’s promoter region and regulates the lncRNA PTCSC3 expression by affecting the binding site of C/EBPα and C/EBPβ (PTCSC3 activators) [98,99,109,110]. PTCSC3 downregulates S100A4, reducing cell motility and invasiveness. Thus, *PTCSC3* mutations could predispose to PTC through the S100A4 pathway [111]. Moreover, *NKX2-1* mutation (c.1016C>T, p.A339V) was observed in a family with multinodular goiter and papillary thyroid cancer [87], but this was not confirmed by another FNMTC study [112].

#### 3.2.3. *NRG1*

*NRG1* polymorphisms produced an association signal in GWAS for thyroid cancer. NRG1 is highly expressed in the thyroid and participates in cell growth pathways, mainly via erb-b2 receptor tyrosin kinase (ERBB)/MAPK [113]. However, NRG1 expression is detected in follicular adenomas, suggesting they are linked to thyroid tumorigenesis [12].

#### 3.2.4. *DIRC3*

Polymorphisms in the *DIRC3* (disrupted in renal carcinoma 3) gene have also been found in thyroid cancer GWAS [12,102,103]. *DIRC3* codifies a lncRNA that was first associated with renal cancer, suggesting a tumor suppressor role [101]. *DIRC3* and *IGFBP5* (insulin-like growth factor binding protein 5) tumor suppressors are within the same topologically associated domain. Moreover, it was observed that *DIRC3* depletion induces an increased *SOX10* (SRY-box transcription factor 10) repression of *IGFBP5* in melanoma cell cultures, corroborating the tumor suppressor role of *DIRC3* [114].

In addition, the TT variant of rs966423 (*DIRC3*, g.217445617C>T) has been associated with worse PTC presentation and prognosis. An increased tumor size, staging, lymph node involvement, and overall mortality was observed in the TT-haplotype [115]. In a Chinese series, rs966423 was also correlated to tumor invasion and multifocality [1]. Nevertheless, no difference in these parameters was observed in a Polish series [116].

#### 3.2.5. Polygenic Contribution

Recently, an increased risk for PTC was associated with a cumulative number of deleterious polymorphisms detected in the same patient. Ten different polymorphisms (rs12129938, rs11693806, rs6793295, rs73227498, rs2466076, rs1588635, rs7902587, rs368187, rs116909374, and rs2289261) related to the PTC development were analyzed, and the presence of each of these SNPs increased the risk to PTC. Nevertheless, if a patient harbors all 10 variants at the same time, the risk of developing thyroid cancer is 6.9-fold greater than those with no variants [117].

#### 3.2.6. Telomere Abnormalities

A decade ago, three independent groups observed that relative telomere length (RTL) is shorter in patients with FNMTC [118,119,120]. As telomerase controls the telomere length, one of these groups investigated *TERC* and *hTERT* (which form telomerase) alterations and observed the amplification of *hTERT* in patients’ leukocytes [118]. However, this finding was not confirmed subsequently [119,120]. In recent years, many alterations in the shelterin complex’s genes have been reported. The shelterin complex is formed by six proteins (POT1, ACD, TINF2, TERF1, TERF2, and TERF2IP), and protects the telomere from DDR mechanisms. Along with telomerase, this complex is vital for genomic stability because telomeric ends resemble DNA double breaks. Telomeric repeat binding factor 1 (TERF1, also known as TRF1), telomeric repeat binding factor 2 (TERF2, also known TRF2), and protection of telomeres 1 (POT1) directly recognize TTAGGG repeats. In contrast, adrenocortical dysplasia protein homolog (ACD, also known as TPP1), TERF1-interacting nuclear factor 2 (TINF2, also known as TIN2), and telomeric repeat binding factor 2 interacting protein (TERF2IP, also known as RAP1) form a complex that differentiates telomeres from sites of DNA damage.

*TINF2* mutation was described in a family with melanoma and thyroid cancer predisposition. Functional analysis showed that mutated *TINF2* was unable to activate *TERF2*, resulting in longer telomere lengths. All shelterin complex’s genes were screened in a subsequent 24 families with FNMTC, and two missense variants in *TINF2* and *ACD* genes were found, but only the *ACD* variant was predicted as deleterious [121]. 

Another group reported a new mutation in *POT1* (c.85G>T; p.V29L) [122] in an Italian FNMTC. *POT1* disruptions can interfere with the interaction of the POT1-ACD complex. In agreement with these findings, another *POT1* mutation (c.268A>G, p.K90E) was described in a family with a predisposition to several tumors (melanoma, breast, kidney, and thyroid cancer, pituitary tumor, and Cushing syndrome) [123]. Moreover, an association between the increased risk of thyroid cancer and the presence of an intronic variant of *POT1* (rs58722976) was also observed in a cohort of childhood cancer survivors [124].

Altogether, it suggests that telomere abnormalities and shelterin complex genes alteration may influence the predisposition to the FNMTC.

#### 3.2.7. miRNA

The miRNA-related SNPs affect the microRNA biogenesis and function. A large study evaluated approximately 80 families displaying Mendelian-like inheritance and found two candidate miRNA (*let-7e* and *miR-181b*). The variants of let-7e and miR-182b-2 were located at the 5′ end of 3p mature miRNA and the 3′ end of 5p mature miRNA, respectively, which downregulate the expression by impairing the miRNA processing [125]. The gain or loss of specific miRNAs is an important oncogenic event [69].

### 3.3. Whole Exome/Genome Sequence

The whole-exome sequence (WES) or the whole genome sequence (WGS) of family members with FNMTC is another strategy besides the GWAS in large populations of differentiated thyroid cancer (DTC). Using this approach, an enormous number of variants is detected, demanding some criteria to filter and select the candidate variants. In general, minor allele frequency (MAF), the expression in thyroid and predictor functions (i.e., SIFT, PolyPhen, CADD, and others) are used as filters. Variants related to cancer pathways can also be used as filters. Since the application of this strategy has been consolidated for genetic studies in recent years, some authors have proposed new variants involved in FNMTC. Many are still under validation.

#### 3.3.1. *SRRM2*

The association of linkage analysis and WES identified an *SRRM2* variant in a family with FNMTC [126]. However, this variant was not exclusively present in FNMTC, as it was found in sporadic NMTC cases, implying the occurrence of FNMTC may also depend on environmental factors or other genes [126].

#### 3.3.2. *NOP53*

The presence of rs78530808 (*NOP53*, c.91G>C, p.D31H) was observed in one family with FNMTC when using a less strict filter than other studies (MAF < 2%) [116]. NOP53 participates in ribosome biogenesis and regulates the p53 activation in the case of ribosome biogenesis perturbation. The variant c.91G>C was also identified in three out of 44 families with FNMTC [127]. In the tumor samples, NOP53 expression was increased when compared to the adjacent normal tissue. Furthermore, *NOP53* knockdown inhibited cell proliferation and colony formation in vitro [127]. Altogether, these findings suggested that this variant could have an oncogenic role in thyroid tumorigenesis [127].

#### 3.3.3. *HABP2*

*HABP2* variant is an excellent example to describe how careful we should be with possible false-positive findings. The variant G534E was described in a family with seven members with PTC [128]. However, this finding was severely criticized later by other researchers. Even though it seemed the right candidate in the beginning, further studies did not confirm it in other populations. Furthermore, since its MAF is high in the European population, we would expect a higher incidence of FNMTC [129]. Besides, the prevalence of this same variant was similar among patients with FNMTC, sporadic PTC, and controls [130,131].

### 3.4. Candidate Variants Associated with FNMTC

Recently, different groups have pinpointed a list of candidate variants in FNMTC. A Korean study identified seven candidate variants localized in *ANO7, CAV2, KANK1, PIK3CB, PKD1L1, PTPRF,* and *RHBDD2* genes in a family with four patients with PTC [132]. In addition, a Brazilian group reported seven new variants located in *FKBP10, PLEKHG5, P2RX5, SAPCD1, ANXA3, NTN4*, and *SERPINA1* [133].

In a large series including 17 families with isolated FNMTC and FNMTC associated with other malignancies, 41 rare candidate variants were identified in *TDRD6, IDE, TINF2, RNF213, AGK, NHLH1, TMCC1, ALB, THBS4, C5orf15, KLH3, FGFR4, SMARCD3, GPR107, NSMF, SVIL, EIF3, RNF169, NFRB, CIS, CDH11, EDC4, FOXA3, CDS2, NAPB, SALL4, ATG14, UNC79, LZTR1, ATP13A2, CTDSP1, MAPKAPK3, AARS, KDSR, ZNF302, ZNF17, ITGAD, FGD6, PDPR*, and *EFCAB8* genes. Cancer susceptibility genes (*CHEK2, PRF1, ATM, AKAP13, SLC26A11*) were also observed [11]. As described before, the authors further correlated the presence of *TINF2* (a shelterin gene) to families with PTC and melanoma.

It was also interesting to observe that some of these genes have already been associated with thyroid cancer predisposition [59,134]. Despite these promising findings, most of the variants need to be better investigated for its functional role in thyroid cancer risk.

## 4. Conclusions

It was expected that the advent of new technologies of genome study would shed new light on the genetic predisposition of FNMTC. The NGS certainly did shed light on a whole new spectrum of variants and pointed to the co-occurrence of several variants in FNMTC. However, the limiting point in this scenario is the lack of a detailed in vitro validation that could precisely identify the contribution of each variant for the complex FNMTC entity. Moreover, the expansion of already known genetic data in multiple cohorts is essential to establish their role in FNMTC carcinogenesis.

## Figures and Tables

**Table 1:** Genetic alterations in syndromes related to non-medullary thyroid cancer (NMTC).

Syndrome	Gene	Inheritance Pattern	Other Malignant Tumors	Prevalent Types of Thyroid Tumors	Benign Manifestations	Reference
Ataxia-telangiectasia syndrome *	*ATM*	AR *	Lymphocytic leukemia, lymphoma, stomach adenocarcinoma, medulloblastoma, glioma	FTC, PTC ^•^	degenerative cerebellar atrophy, telangiectasias, immune defects	[15]
	*ATM*	AD	breast cancer, digestive tract cancer, lymphoma, leukemia			[15,16]
Carney complex	*PRKAR1A*	AD	-	Follicular hyperplasia, nodular hyperplasia, FA, cystic changes, PTC, FTC	Spotty skin pigmentation (lips, conjunctiva, vaginal, and penile mucosa), cutaneous and mucosal myxoma, cardiac myxoma, breast myxomatosis, primary pigmented nodular adrenocortical disease, GH-producing adenoma, large cell calcifying Sertoli cell tumors, psammomatous melanotic schwannomas	[17]
Cowden syndrome	*PTEN, SDHB-D, SEC23B, KLLN, PARP4, AKT1, PIK3CA, USF3, TTN, RASAL1*	AD	FTC, breast cancer, epithelial endometrial cancer, colon cancer, renal cell carcinoma melanoma	MNG, Hashimoto thyroiditis, FA, FTC, cPTC, FVPTC, C-cell hyperplasia	Macrocephaly	[18,19]
DICER1 syndrome	*DICER1*	AD	Pleuropulmonary blastoma, ovarian Sertoli-Leydig cell tumor, genitourinary and cerebral sarcomas	MNG, PTC, FA	MNG, cystic nephroma	[20,21]
Familial adenomatous polyposis	*APC*	AD	Digestive tract cancers, fibrosarcomas	CMVPTC, PTC	Intestinal polyps, osteomas, fibromas, desmoid tumors, dental abnormalities, leiomyomas, congenital hypertrophy of the retinal pigment epithelium	[22,23]
Li-Fraumeni syndrome	*TP53*	AD	Breast, brain, and adreno cortical cancers and sarcomas	cPTC, FVPTC		[13,24]
Werner syndrome	*WRN*	AR	Atypical melanoma, bone, or soft tissue sarcomas	FTC, PTC, ATC	Aging, bilateral cataract, type 2 diabetes mellitus, hypogonadism, meningioma	[2,25]

* Ataxia-telangiectasia syndrome occurs only in autosomal recessive pattern. However, heterozygotic carriers have an increased risk to cancer radio ionizing-induced. ^•^ An increased risk for thyroid cancer was observed in relatives of A-T patients, but the histological type was not specified in those epidemiological analysis. The above information is inferred from susceptibility thyroid cancer studies [26,27].

**Table 2:** Loci and genes associated with non-syndromic familial non-medullary thyroid cancer (FNMTC).

Loci/Gene	Localization	Characteristics	Reference
**Linkage analysis**			
TCO	19p13.2	Oxyphilic PTC	[6]
NMTC1	2q21		[9]
PRN1	1q21	Papillary renal cancer	[8]
MNG1/*DICER1*	14q32	MNG	[7]
**Linkage analysis and NGS**			
*SRGAP1*	12q14		[88]
	8p23.1–p22		[10]
	6q22		[89]
lncRNA inside TG	8q24	Melanoma in 1 family	[90]
Enhancer associated with POU2F1 and YY1	4q32		[91]
**Other methodology**			
*NKX2-1*	14q13.3		[92]

**Table 3:** Genes associated with genetic predisposition of sporadic papillary thyroid cancer.

Locus	Nearest Gene	Population	Reference
9q22.33	*FOXE1, PTCSC2*	Belarus, Iceland, Italy, Korea, Netherlands Poland, Spain, USA	[98,99,100]
14q13.3	*PTCSC3, NKX2-1, MBIP1*	Iceland, Italy, Korea, Netherlands, Poland, Spain, USA	[98,99]
2q35	*DIRC3*	Iceland, Italy, Korea, Netherlands, Poland, Spain, UK, USA	[99,101]
8p12	*NRG1*	Iceland, Korea, Netherlands, Spain, USA	[99,102]
1q42.2	*PCNXL2*	Iceland, Korea, Netherlands, Spain, USA	[102,103]
**European Only**			
3q26.2	*LRRC34*	Iceland, Netherlands, Spain, USA	[103]
5p15.33	*TERT*	Iceland, Netherlands, Spain, USA	[103]
5q22.1	*EPB41L4A*	Iceland, Netherlands, Spain, USA	[103]
10q24.33	*OBFC1*	Iceland, Netherlands, Spain, USA	[103]
15q22.33	*SMAD3*	Iceland, Netherlands, Spain, USA	[103]
**Korean Only**			
12q14.3	*MSRB3*	Korea	[102]
1p13.3	*VAV3*	Korea	[102]
4q21.1	*SEPT11*	Korea	[102]
3p14.2	*FHIT*	Korea	[102]
19p13.2	*INSR*	Korea	[102]
12q24.13	*SLC8B1*	Korea	[102]

## Abbreviations

AD	autosomal dominant
AR	autosomal recessive
ATC	anaplastic thyroid cancer
CMVPTC	cribriform-morular variant of PTC
cPTC	classical PTC
DDR	DNA damage response
DTC	differentiated thyroid cancer
FA	follicular adenoma
FAP	familial adenomatous polyposis
FNMTC	familial nonmedullary thyroid cancer
FTC	follicular thyroid cancer
FVPTC	follicular variant of PTC
GWAS	genome-wide association studies
HR	hazard ratio
LOD	logarithm of odds
MAF	minor allele frequency
miRNAs	microRNAs
MNG	multinodular goiter
MTC	medullary thyroid cancer
NGS	next generation sequencing
NMTC	nonmedullary thyroid cancer
PTC	papillary thyroid cancer
SNPs	single nucleotide polymorphisms
TCGA	The Cancer Genome Atlas
VUS	variant of uncertain significance
WES	whole exome sequence
WGS	whole genome sequence
